# Extraction of well-fixed extended porous-coated cementless stems using a femoral longitudinal split procedure

**DOI:** 10.1007/s12570-015-0322-2

**Published:** 2015-08-29

**Authors:** Satoshi Nagoya, Mikito Sasaki, Mitsunori Kaya, Shunichiro Okazaki, Kenji Tateda, Toshihiko Yamashita

**Affiliations:** Department of Musculoskeletal Biomechanics and Surgical Development, Sapporo Medical University, Sapporo, Japan; Department of Orthopedic Surgery, Sapporo Medical University, South 1 West 16, Chuo-Ku Sapporo, 060-8543 Japan

**Keywords:** Extraction of femoral stem, Removal of stem, Split osteotomy, Hip joints, Extended porous coated, Total hip arthroplasty

## Abstract

We present a technique of posterior femoral longitudinal split (FLS) osteotomy. This technique allows the expansion of the metaphyseal–diaphyseal region of the proximal femur facilitating extraction of well-fixed extended porous-coated stems. The extractions were performed using extended transfemoral osteotomy (ETO) and FLS osteotomy between June 2002 and March 2014. The study group, which comprised patients with well-fixed extended porous-coated stems, consisted of two men and ten women with an average age of 63.2 years. The stem was successfully removed using the FLS procedure in 8 of the 10 hips. Reimplantation surgery was performed in 6 of the 12 hips with ARMD, periprosthetic infection, or metallosis. This FLS technique may allow the easy removal of well-fixed extended porous-coated stems and become an alternative method for the removal of all stems.

Recently, there has been a shift toward the use of cementless THAs in place of cemented THAs. However, several complications exist in terms of articulation as well as the stem and acetabular components. Although problems associated with articulation failures can be resolved by articulation exchange, some dislocations or periprosthetic infections may require the complete removal of the implant.

In general, well-fixed cementless stems have been difficult to remove. Cementless stems osseointegrated with the proximal femur can be extracted by insertion of a thin osteotome between the proximal femoral cortex and the cemetless stem. However, in patients treated with an extended porous-coated cementless stem, in which the stem is firmly osseointegrated with the femur, a thin osteotome cannot reach the osseointegrated portion distal to metaphysis of the femur, making extraction of the stem extremely difficult.

In 1995, Younger et al. [1] reported a new extraction method for a well-fixed cementless and/or cemented femoral stem. In this process, referred to as extended transfemoral osteotomy (ETO), the lateral femoral fragment is longitudinally opened to visualize the whole stem, and the stem is then removed. Although, in theory, this method facilitates the smooth removal of the stem implant allowing the proximal lateral femoral fragment to be reduced to its original position with cerclage wiring at reimplantation, it involves the risk of proximal migration of the proximal femoral fragment. Configuration of the proximal femur should, therefore, be preserved for successful revision surgery.

We found that distally well-fixed stems could be successfully and simply removed by gently striking after the preparation of a longitudinal split line along the posterior aspect of the femoral bone during the initial ETO process.

We explored the possibility that a safe and easy extraction procedure for the removal of well-fixed extended porous-coated cementless stems, such as AML stems, could be developed. The purpose of this paper is to introduce a useful extraction procedure for well-fixed femoral stems and report the clinical results of this procedure.

## Materials and methods

We experienced 111 hips that required removal of the femoral stem as part of revision surgery from 1999 in our department. Of the 111 hips, 92 were cemented stems that were loosened, and these stems were relatively easy to remove, despite difficulties with cement extraction. Nineteen hips required the removal of cementless stems, and 12 hips had extended porous-coated stems. The study group, which comprised the patients with well-fixed extended porous-coated stems, consisted of two men and ten women with an average age of 63.2 years (range 42 to 94 years) (Table 1). This study was approved by the university and hospital institutional review board (IRB).

**Table 1:** Patient demographic data

Case	Gender	Age	Side	Stem		Acetabular		Head		Articulation	Cause of removal
				Type	Fixation	Cup	Insert	Composition	Size		
1	male	46	L	AML plus	ingrown	Duraloc	conv poly enduron	CoCr	22.225 mm	MOP	late infection
2	female	64	L	AML 5/8	ingrown	Duraloc	conv poly enduron	CoCr	22.225 mm	MOP	late Tbc
3	female	53	L	AML plus	ingrown	Duraloc	conv poly enduron	CoCr	22.225 mm	MOP	ARMD
4	female	61	R	AML plus	ingrown	Duraloc	conv poly enduron	CoCr	22.225 mm	MOP	late infection
5	female	61	L	full coated stem	ingrown	Duraloc	conv poly enduron	CoCr	22.225 mm	MOP	late infection
6	female	65	L	solution	fibrous stable	fin cementless	conv poly enduron	CoCr	22.225 mm	MOP	metallosis
7	male	42	L	Anatomic	ingrown	Zimmer jumbo	conv poly enduron	CoCr	32 mm	MOP constrained	late infection
8	female	70	R	AML plus	ingrown	Duraloc	constrained poly	CoCr	22.225 mm	MOP	ARMD, neck fracture
9	female	52	L	AML plus	ingrown	Duraloc	conv poly enduron	CoCr	22.225 mm	MOP	late infection
10	female	82	L	AML plus	ingrown	Pinaccle	Ultamet	CoCr	36 mm	MOM	ARMD
11	female	68	L	AML Replica	ingrown	Duraloc	conv poly enduron	CoCr	28 mm	MOP	ARMD
12	female	94	R	AML plus	ingrown	Duraloc	conv poly enduron	CoCr	28 mm	MOP	neck fracture

*MOP* metal-on-polyethylene, *MOM* metal-on-metal

The 12 extended porous-coated stems consisted of 7 AML Plus stems (DePuy), one AML 5/8 stem (DePuy), one Solution revision stem (DePuy), one Replica stem (DePuy), one Zimmer Versys full-porous coat stem (Zimmer, Warsaw, IN ), and one fully porous-coated stem of unknown manufacture. All of these stems were categorized as extended porous-coated stems [3]. Articulation and acetabular cup, as well as the cause of revision, are described in Table 1.

## Operative procedure

**Fig. 1:**
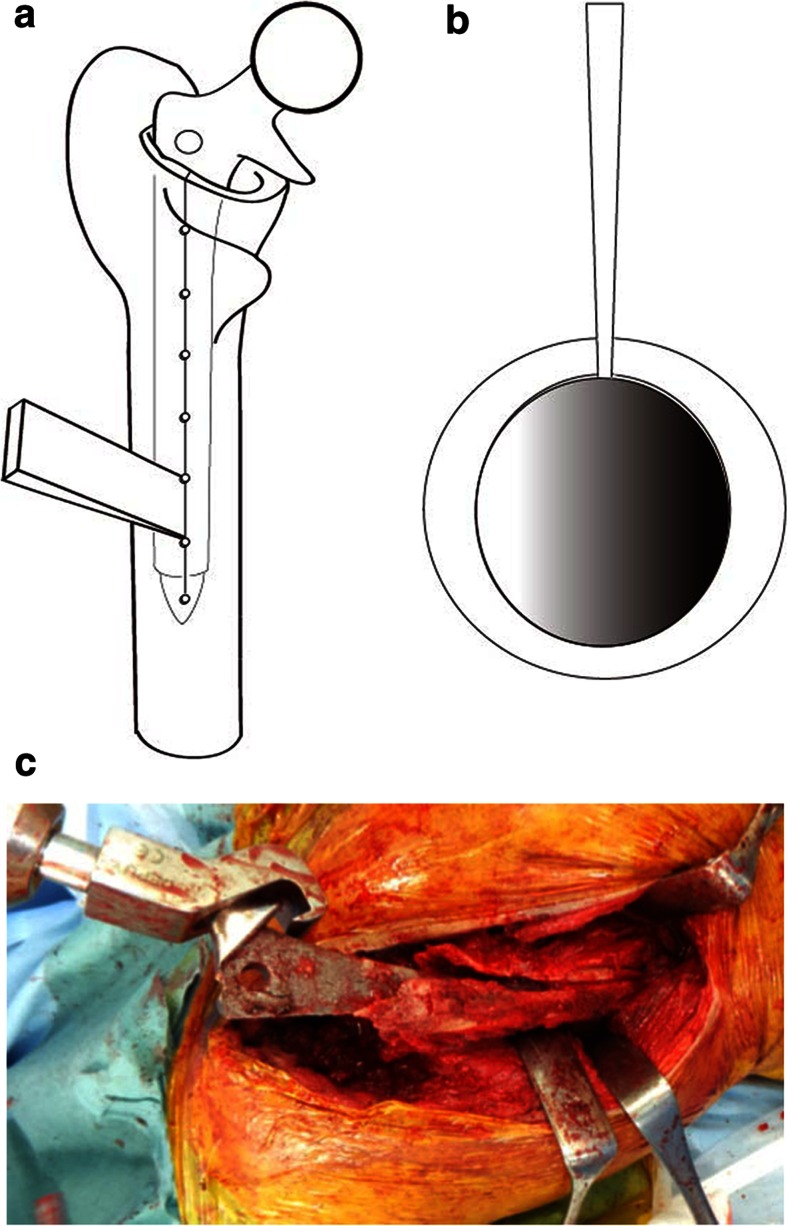
Diagram showing the femoral longitudinal split (FLS) procedure. Multiple drill holes were connected by an osteotome at the posterior aspect of the proximal femur (**a**). Diagram showing the inserted osteotome twisted to open the split portion (**b**). Diagram showing the FLS procedure applied to case 9 (**c**)

With the patient in the lateral decubitus position, the hip joint was dislocated through a posterolateral approach, the posterior aspect of the femur was exposed, and the vastus lateralis muscle was detached from the intertrochanteric eminentia to expose the femoral linea aspera corresponding to the length of the implanted femoral stem. A Kirschner wire was used to drill multiple small holes (2.0 mm in diameter) at 1-cm intervals longitudinally along the exposed posterior linea aspera to the tip of the femoral stem, and these small holes were connected by osteotome to form a femoral longitudinal split (FLS). Prophylactic cerclage wire was applied at the distal portion of the split osteotomy to prevent femoral fracture. At a point along the split, two-thirds distal from the femoral head, the inserted osteotome was twisted to open the split portion (Fig. 1). At this stage, local debonding of the integrated bone from the porous surface of the stem could be obtained, and simple striking of the stem in a proximal direction made it possible to remove the well-fixed stem. In cases where removal in this manner is impossible, debonding could be achieved by the insertion of a thin osteotome, allowing the stem to be removed.

## Results

**Fig. 2:**
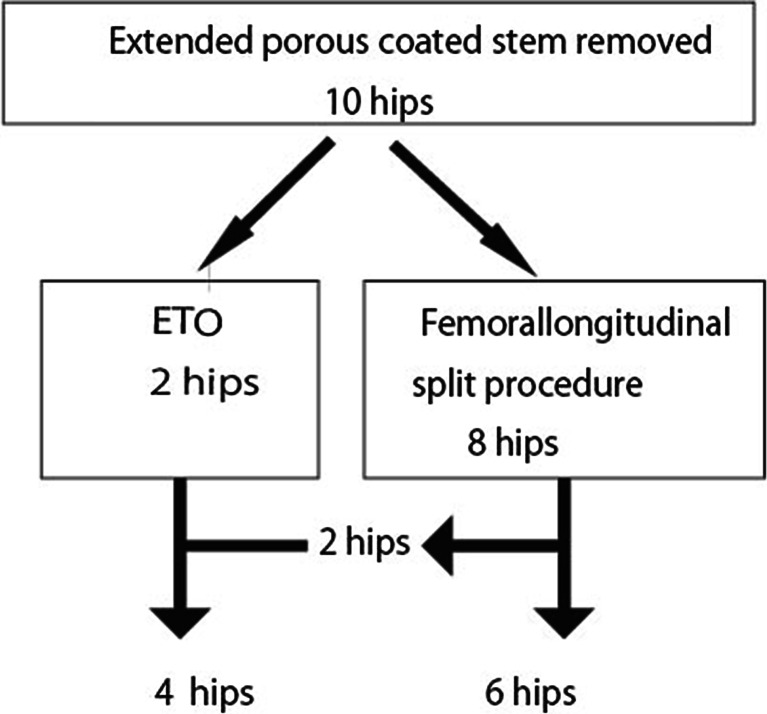
Flow chart of the removal procedure for an extended porous-coated stem

We used the FLS procedure to extract extended porous-coated stems in 10 of the 12 hips. The remaining two hips were treated before 2004, and ETO was initially planned. The stem was successfully removed using the FLS procedure in 8 of the 10 hips (Fig. 2). As two hips in one patient who experienced late infection of the THA resulting from rheumatoid arthritis could not be removed using the FLS procedure, we eventually decided upon ETO. Fractures occurred in 5 hips during the operation. One patient had a transverse femoral fracture after ETO. One patient experienced a right transverse femoral fracture and left trochanteric fracture, resulting in the FLS being converted to ETO. The other two hips had trochanteric fractures during the FLS. One hip experienced a trochanteric fracture during the observation period for resolution of infection, and the other had a trochanteric fracture after periprosthetic infection.

Evaluation of the biological fixation of the stem revealed that 11 hips had bone ingrown and one hip had fibrous stable fixation. Three hips displayed greater than grade 2 stress shielding. All hips had canal filling except for the hip with fibrous stable fixation. In the two hips requiring conversion of the FLS procedure to ETO, remarkable stress shielding of the proximal femur was observed.

The FLS procedure for extraction of the stems was performed between November 2007 and December 2014. The average duration from initial implantation to extraction surgery was 85.5 ± 33.7 months. Average bleeding during operation was 336 ml (110–620 ml), and average operation time was 151 min (Table 2).

**Table 2:** Data for patients treated by ETO who were excluded in order to focus on FLS

Case	Primary THA year	Year at extraction	Interval to extraction (months)	Op. time (min)	Intraop. bleeding (ml)	Stem extraction methods	Periop. complications	Reimplantation
3	1999	2007	95	117	620	FLS	None	Successful
6	2005	2012	100	183	280	FLS	None	Successful
7	2008	2013	60	95	110	FLS	Trochanteric fracture (infected)	Planned
8	2003	2013	121	139	500	FLS	Large tissue defect	Successful
9	2005	2013	97	186	400	FLS	Trochanteric fracture (infected)	Successful
10	2008	2014	70	158	150	FLS	None	Planned
11	2012	2014	21	151	350	FLS	None	Successful
12	2004	2014	120	180	280	FLS	None	Planned
Average			85.5	151	336			

Reimplantation surgery was performed in 6 of the 12 hips, among which two hips had ARMD, two hips had periprosthetic infection, 1 hip had metallosis in the impingement between the Ti cup and CoCr head, and one hip had a stem neck fracture. Three other hips are scheduled for revision surgery. The remaining three hips were not eligible for reimplantation due to residual persistent osteomyelitis or decline in general health due to liver cirrhosis (Table 2).

With regard to the reimplantation of stems, athough one patient with ETO was treated with a longer cementless MP stem (Link), cementless stems that were shorter or of equal length to the extracted stem were usually implanted in patients treated by the FLS procedure with support of cerclage wire. Alloclassic stems (Zimmer) were implanted in 2, SL Plus MIA stems (Smith & Nephew Orthopaedics) in 2 and an MP system (Link) in 1 patient.

With regard to articulation, conventional polyethylene was used in two hips, and a constrained cup was used in the other 4 hips: one with huge bone and soft tissue defects resulting from multiple debridements due to difficulties in eradicating periprosthetic infection, one with extensive soft tissue loss including the gluteus medius and minimus muscles due to ARMD, one with a bone defect of the greater trochanter due to late periprosthetic infection, and one in which a comminuted femoral fracture occurred during extraction by ETO (Table 2). Although there was one dislocation (case 9), no loosening or migration of the stem was observed. Further, there was no recurrence of periprosthetic infection or symptomatic venous thromboembolism. Reimplantation was performed as second-stage operation in all but one patient (case 6).

## Discussion

We herein described a useful method, referred to as the femoral longitudinal split (FLS) procedure, for the extraction of well-fixed extended porous-coated cementless stems while preserving the normal configuration of the proximal femur. Using the FLS procedure, we achieved the successful extraction of the femoral stem in 8 out of 10 patients who had extended porous-coated cementless femoral stems.

The extracted stem was an extended porous-coated stem in each case. Although one hip had fibrous stable fixation, 11 hips were evaluated radiographically with bone ingrown fixation. When removing osseointegrated cementless stems, debonding of the stem from the surrounding bone using a thin osteotome can be difficult. ETO offers a method for extraction in which the lateral femoral fragment is opened longitudinally to visualize the whole stem, followed by the use of a thin or wire osteotome to debond and remove the stem [1, 2]. In contrast, our method, based on the FLS procedure, affords a minimally invasive stem extraction procedure in which the posterior aspect of the proximal femur is split.

**Fig. 3:**
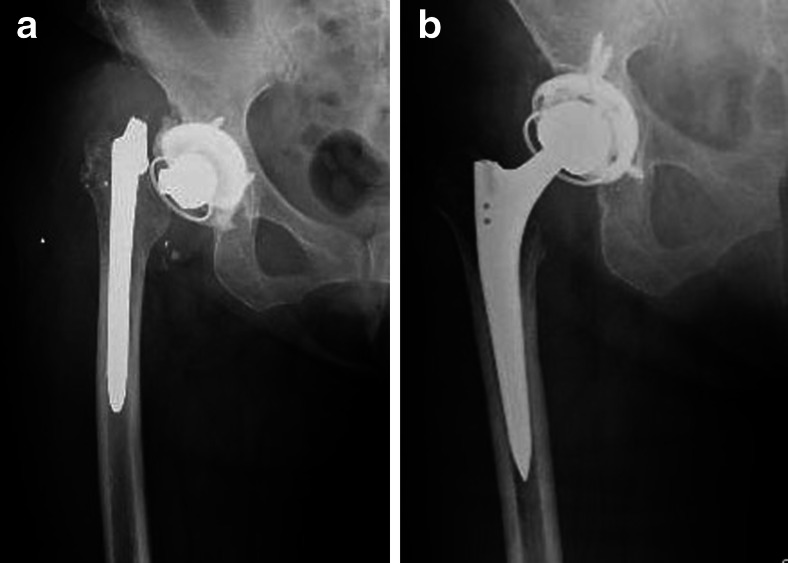
Radiograph of a 70-year-old female showing a femoral stem fracture after multiple dislocations of a THA (**a**) and successful reimplantation with a cementless stem was performed (**b**)

Jack et al. [3] reported satisfactory clinical results for 18 patients who underwent revision THA with slot femoral osteotomy while leaving the most proximal metaphysis intact. In this study, however, 80 % of the extracted stems were curved anatomic stems fixed in the metaphysis. Bauze [4] also reported posterior longitudinal split osteotomy for femoral component extraction in revision total hip arthroplasty. However, these authors did not include extended porous-coated stems [5]. In contrast, the fact that almost of the stems in our series were distally osseointegrated extended porous-coated stems suggests that a more extensive split osteotomy through the proximal part of the femur to facilitate extraction of the stem was required. In our method, a Kirschner wire was used to drill multiple small holes (2.0 mm in diameter) at 1-cm intervals longitudinally along the exposed posterior linea aspera to the femoral stem, and these small holes were connected by an osteotome to make a femoral longitudinal split (Fig. 1). This process did not leave any longitudinal bone defects in the femur. The use of tight cerclage wiring, consisting of ultrahigh molecular weight polyethylene fiber cable [6], to prevent femoral cracking did not produce any adverse effects, such as diminished femoral diameter. Furthermore, this method did not interrupt further reimplantation surgery as the configuration of the proximal femur was conserved (Fig. 3).

At present, the choice of stem retention or extraction in patients facing any of a variety of problems associated with well-fixed extended porous-coated stems is based on a number of different treatment strategies employed to resolve these problems. Indeed, even in cases of periprosthetic infection, hip surgeons tend to retain the well-fixed extended porous-coated stem because of difficulties associated with its extraction [7] and the potential for catastrophic failure, such as comminuted femoral fracture, when violent extraction methods are used. However, periprosthetic infection, in which a biofilm [8] may be produced, can be difficult to resolve. Furthermore, in cases of ARMD, tissue necrosis might spread, with allergic reactions to CoCr ions resulting in catastrophic failure; thus, removal of the CoCr element must be considered as the first treatment option [9, 10]. Minimal damage to the bone during the stem extraction process can make stem extraction possible even in these severe situations.

Limitations to this study are that not all patients underwent reimplantation surgery and that only a small number of cases were analyzed. Further, this study was retrospective and did not have a control group.

In the reimplantation process, ETO required relatively longer stems that reached beyond the osteotomy to achieve initial stability of the stem. However, it was sufficient for the reimplanted stems to be shorter or equal in length to the extracted stem in the FLS procedure as the fixation reliably restored the integrity of the metaphysis. In the FLS procedure, cerclage wiring is speculated to be sufficient to prevent comminuted fracture as no large bone defects remain.

Although this technique allows the expansion of the metaphyseal–diaphyseal region of the proximal femur facilitating extraction of well-fixed extended porous-coated stems, the fact that fractures could not be prevented during operation indicated that there was learning curve of this surgical procedure. Even if this process was not sufficient to remove the stem, it is supposed that our procedure can easily be converted to conventional ETO that might be reliable to extract stems and avoid femoral fractures. This useful FLS procedure may allow the easy removal of well-fixed extended porous-coated stems and become an alternative method for the removal of all stems.

## References

[1] Younger TI, Bradford MS, Magnus RE (1995). Extended Proximal Femoral Osteotomy A New Technique for Femoral Revision Arthroplasty. J Arthroplasty.

[2] Mardones R, Gonzalez C, Cabanela ME (2005). Extended Femoral Osteotomy for Revision of Hip Arthroplasty. Results and Complications. J Arthroplasty.

[3] Jack CM, Molloy DO, Esposito C (2013). Limited slot femorotomy for removal of proximally coated cementless stems. A 10-year follow-up of an unreported surgical technique. J Arthroplasty.

[4] Bauze AJ, Charity J, Tsiridis E (2008). Posterior longitudinal split osteotomy for femoral component extraction in revision total hip arthroplasty. J of Arthroplast.

[5] Khanuja HS, Vakil JJ, Goddard MS (2011). Cementless Femoral Fixation in Total Hip Arthroplasty. J Bone Joint Surg Am.

[6] Oe K, Jingushi S, Iida H, Tomita N (2013). Evaluation of the clinical performance of ultrahigh molecular weight polyethylene fiber cable using a god osteosynthesis model. Biomed Mater Eng.

[7] Lee YK, Lee KH, Nho JH (2013). Retaining well-fixed cementless stem in the treatment of infected hip arthroplasty. Good results in 19 patients followed for mean 4 years. Acta Orthop.

[8] Nguyen LL, Nelson CL, Saccente M (2002). Detecting bacterial colonization of implanted orthopaedic devices by ultrasonication. Clin Orthop.

[9] Lombardi AV, Barrack RL, Berend KR (2012). J Bone Joint Surg Br.

[10] Pelt CE, Erickson J, Clarke I (2013). Histologic, serologic, and tribologic findings in failed metal-on-metal total hip arthroplasty: AAOS exhibit selection. J Bone Joint Surg Am.

